# Chirality Enhancement Using Fabry–Pérot-Like Cavity

**DOI:** 10.34133/2020/7873581

**Published:** 2020-02-28

**Authors:** Jiaxin Bao, Ning Liu, Hanwei Tian, Qiang Wang, Tiejun Cui, Weixiang Jiang, Shuang Zhang, Tun Cao

**Affiliations:** ^1^School of Optoelectronic Engineering and Instrumentation Science, Dalian University of Technology, Dalian 116024, China; ^2^State Key Laboratory of Millimeter Waves, School of Information Science and Engineering, Southeast University, Nanjing 210096, China; ^3^School of Physics & Astronomy, University of Birmingham, Birmingham, B15 2TT, UK

## Abstract

Chiral molecules that do not superimpose on their mirror images are the foundation of all life forms on earth. Chiral molecules exhibit chiroptical responses, i.e., they have different electromagnetic responses to light of different circular polarizations. However, chiroptical responses in natural materials, such as circular dichroism and optical rotation dispersion, are intrinsically small because the size of a chiral molecule is significantly shorter than the wavelength of electromagnetic wave. Conventional technology for enhancing chiroptical signal entails demanding requirements on precise alignment of the chiral molecules to certain nanostructures, which however only leads to a limited performance. Herein, we show a new approach towards enhancement of chiroptical effects through a Fabry–Pérot (FP) cavity formed by two handedness-preserving metamirrors operating in the GHz region. We experimentally show that the FP cavity resonator can enhance the optical activity of the chiral molecule by an order of magnitude. Our approach may pave the way towards state-of-the-art chiral sensing applications.

## 1. Introduction

Chirality plays a significant role in chemistry and biology, and it dictates how pharmaceuticals, additives, pesticides, or agrochemicals, just name a few, are combined [1, 2]. The chiroptical response of natural materials, however, is inherently low since the chiral molecule's size is much smaller than the wavelength of electromagnetic (EM) wave [3]. Recently, it shows that locally varying the distribution of the EM field may enhance the chiroptical effect of the weak chirality [4, 5]. Since then, many works have been devoted to exploring various structures that provide localised EM fields for enhancing the chiral signals, such as antennas, [6] plasmonic structures [7–12], and dielectric particles [13, 14]. For example, chiral metallic nanostructures have been applied to enhance the chirality in proteins [15]. These techniques may result in key practical applications, whereas the geometrical complexity has yet forbidden a quantitative comparison with theory [5]. Recently, optical cavity is shown to be an efficient scheme for detecting a single molecule since the resonant recirculation of a beam within the cavity enables the light to sample the molecule many times [16–20]. For example, in an optical waveguide detector, the input beam can only interact with the target molecule one time. In contrary, by using an optical cavity with a quality factor of 10^8^, the molecule was sampled ~10^5^ times [21]. Moreover, the development of optical cavity made of microtoroid whispering-gallery resonators was proposed to increase the sensitivity of the biodetector [22]. Our work extends this strategy by introducing a chiral Purcell effect through which the optical cavity can enhance chiroptical signals emitted from the biomolecules [3, 23]. Likewise, the chiral response can be increased by the cavity possessing chiral-symmetric geometry, leading to a unidirectional lasing radiation in the far field [3]. However, these methods rely on the precise positioning of chiral molecules to these nanoscale particles.

Large-scale cavities such as Fabry–Pérot (FP) cavities have been widely used for enhancing the interaction between light and matters. In comparison with the localized resonances, the FP cavity has the advantage of position insensitivity. However, conventional FP cavities cannot be used for chirality enhancement because the reflection of light by the mirrors flips the handedness of light. As a result, the chiral responses of the forward propagating light are canceled by the backward propagating light inside the FP cavity [24]. Metamaterials are artificial materials engineered to realise a plethora of novel functionalities and phenomena, which are unreachable by naturally occurring materials [25–30]. They are usually arranged in periodic patterns, at a scale which is much less than the wavelength of interest [31, 32]. Recent progress in the field of 2D metamaterials (metasurfaces) has led to superior control over wave propagation [33], with numerous examples of device applications, such as metalenses [34], vortex generation [35], computer-generated holography [36], and programmable coding [37]. In particular, it has been shown that metasurfaces can be engineered to arbitrarily manipulate the polarization state of light upon transmission or reflection [38–40]. For example, metasurface composed of split-ring meta-atom [41] possesses a birefringence that is suitable for polarization conversion [39, 42–46], which has been widely explored in the GHz region. An ultrathin metasurface polarimetric device is demonstrated to obtain high-efficiency and broadband linear polarization conversion in the THz region [47]. Broadband metasurface circular polarizers are observed in the optical region using stacked nanorod arrays with a rotational twist [48] and gold helix resonator [49]. In particular, with a suitable design of the metasurfaces, it is possible to preserve the handedness of light upon reflection [50].

In this work, we exploit the FP cavity formed by a pair of metal/dielectric/metal trilayered handedness-preserving metasurfaces (HPMs) for enhancing light-chiral element interaction. As a proof of concept, we consider the enhancement of chirality for a metamaterial consisting of bilayer twisted cross wires which exhibit very weak chiral responses at the frequencies of interest. Each HPM consists of an array of cutting-wire antennas patterned on the F4B dielectric layer integrated with a ground Cu grid array. The two HPMs forming the FP cavity are perpendicular to each other. The use of HPMs allows the reflected waves to maintain their handednesses and continue to constructively accumulate the chiral responses. The two ground Cu grid films work as mirrors to reflect the majority of EM wave that propagate out of the FP cavity, while still letting a portion of the EM wave propagate into the cavity. This configuration greatly enhances the optical rotation dispersion (ORD) and enables the polarization rotation angle of the bilayer twisted cross wires to be one order larger in magnitude than that without the cavity. Both the co- and cross-polarization transmissions are measured to verify the chiral enhancement. Thus, our findings offer a new design principle for next-generation resonator-enhanced chiroptical spectroscopy that can sense a small quantity of chiral molecule.

## 2. Results

**Figure 1:**
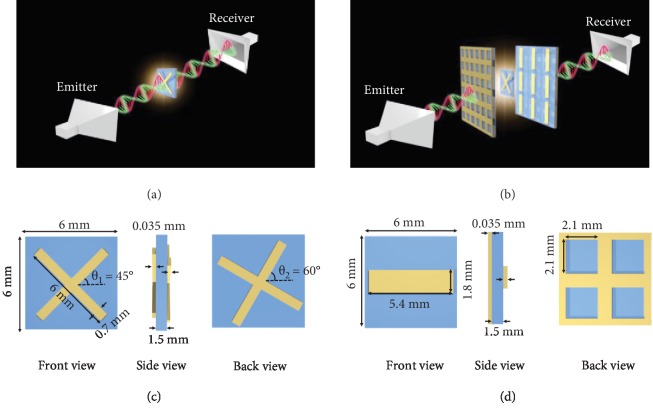
Design schematics of the (a) pure chiral molecule, (b) chiral molecule placed inside a FP cavity formed by two metamirrors, (c) chiral metamolecule, and (d) metamirror, wherein the geometrical parameters of the structures are indicated. Each Cu grating has a length of 5.4 mm, a width of 1.8 mm, a height of 0.035 mm, and a pitch of 6 mm. The height of the Cu mesh plane is 0.035 mm. The F4B boards have a thickness of 1.5 mm. Note, in the experiment, we measure an array of chiral structures instead of a single one.

Figures 1(a) and 1(b) schematically show the measurement of optical activity of a chiral metamolecule alone and a chiral metamolecule placed inside a FP cavity formed by two metamirrors, respectively. The comparison between the two measurements can provide the information on the effectiveness of the FP cavity on enhancing the power of chirality sensing. The chiral metamolecule consists of a pair of 0.035 mm thick Cu cross wires twisted relative to each other by an angle ranging from *θ*_1_ = 45° to *θ*_2_ = 60° and separated by a F4B dielectric layer of thickness 1.5 mm (Figure 1(c)). The length and width of the Cu wire are 6 mm and 0.7 mm, respectively. The FP cavity is formed by two metamirrors (Figure 1(b)). The geometry of the unit cell of the metamirror is shown in Figure 1(d). The grating can block the copolarized waves while transmitting the cross-polarized waves. The size of each grid is 2.1 × 2.1 mm^2^. The length of the FP cavity is 12 mm.

**Figure 2:**
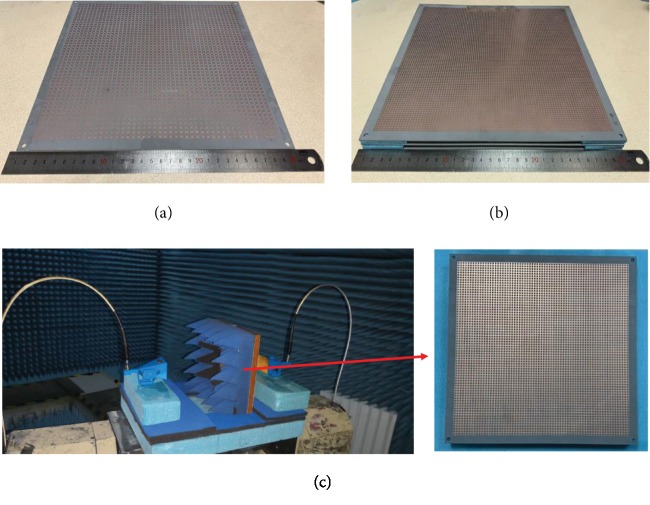
The photographs of the fabricated (a) pure chiral metamolecules and (b) FP cavity configuration structures. (c) Experimental setup in a microwave anechoic chamber for the measurement of the fabricated structures. The inset shows the sample placed inside the compartment.

To experimentally verify our strategy, the designed chiral metamolecule and FP cavity structures are fabricated through printed circuit board (PCB) process. The photographs of the fabricated structures are shown in Figures 2(a) and 2(b). The dimensions of the structures are provided in the schematic picture (Figure 1). As seen in Figure 2(a), a 46 × 46 array Cu cross-wire is patterned on a double side Cu-clad F4B board. Figure 2(b) shows the two reflectors consisting of the Cu gratings and Cu grid layers on both sides of two blank F4B boards and the Cu gratings in two reflectors are parallel to *x*- and *y*-axes, respectively. The eight polymer foam spacers with 5.25 mm thickness are used to support the three F4B boards. The sizes of both structures are 300 × 300 mm^2^. The transmission of the structures is measured using an in-house electromagnetic test platform that is built in the electromagnetic (EM) anechoic chamber with the advantage of low external noise [51]. The EM test platform consists of a pair of Ku-band standard gain pyramid horn antennas and a two-port vector network analyzer. The two standard gain horn antennas are employed as receiving and transmitting devices, and the two-port vector network analyzer is utilized as an EM signal generator and post-processing device, as shown in Figure 2(c). A detailed description of the measurement setup can be found in Materials and Methods.

**Figure 3:**
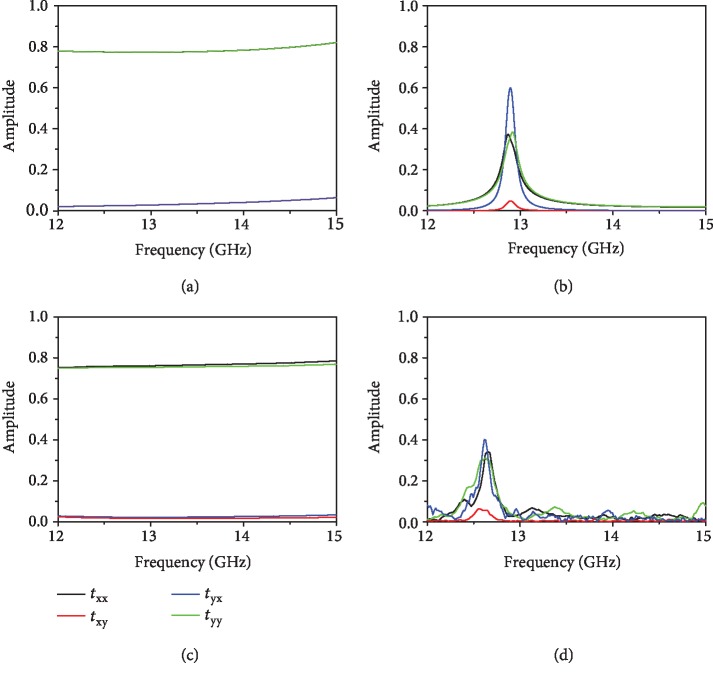
The CST-simulated linear transmission coefficients *t*_*yx*_, *t*_*xy*_, *t*_*xx*_, and *t*_*yy*_ for (a) pure chiral metamolecule and (b) FP cavity configuration structures with *θ*_1_ = 45° and *θ*_2_ = 60°. The measured *t*_*yx*_, *t*_*xy*_, *t*_*xx*_ and *t*_*yy*_ for (c) pure chiral metamolecules and (d) FP cavity configuration structures with *θ*_1_ = 45° and *θ*_2_ = 60°.

Firstly, a commercial 3D full-wave solver (CST MICROWAVE STUDIO®) based on the finite integration technique is performed to compute the chiral response of the layer of metamolecules [52]. Periodic boundary conditions are adopted along the *x*- and *y*-axes, while absorbing boundary condition is used along the *z*-axis that is open (add space) for input and output EM waves. Two orthogonal linearly polarized incident waves propagating along the +*z* axis are used for excitation. In the model, the relative permittivity of F4B is set as 2.2, the loss tangent (*δ*), and conductivity of Cu are 0.001 and 5.8 × 10^7^ S/m accordingly [53]. In Figure 3(a), we present the numerically simulated transmission coefficients of *t*_*xx*_, *t*_*yx*_, *t*_*yy*_, and *t*_*xy*_for the layer of chiral metamolecule, where *t*_*xx*_,*t*_*yx*_, *t*_*yy*_, and *t*_*xy*_ correspond to *x* (input)-to-*x* (output), *x* (input)-to-*y* (output), *y* (input)-to-*y* (output), and *y* (input)-to-*x* (output) polarized transmission conversion efficiencies [54]. The two cross-polarized transmissions of *t*_*yx*_ and *t*_*xy*_ carry less than 5% of the incident power, and the copolarized transmissions of *t*_*xx*_ and *t*_*yy*_ are around 80% in the spectra ranging from 12 to 15 GHz. The chiral metamolecule has a very weak optical activity as indicated by the small cross-polarized transmission (off-diagonal elements of the transmission matrix) [55]. To enhance the chiroptical response of the metamolecule, we position the chiral molecules inside the FP cavity formed by two metamirrors (Figure 1(b)). For the cavity configuration, a resonant peak is observed at the frequency of *f* = 12.9 GHz in *t*_*xx*_,*t*_*yx*_, *t*_*yy*_, and *t*_*xy*_ curves, respectively. As was shown in Figure 3(b), the copolarization transmission coefficient *t*_*xx*_of *x*-polarized wave almost coincides with *t*_*yy*_of *y*-polarized wave. On the contrary, the cross-polarization transmission coefficient *t*_*yx*_ is very different from *t*_*xy*_. Particularly, *t*_*yx*_ shows a peak value of 0.6 and the *t*_*xy*_ is below 0.1 at *f* = 12.9 GHz, indicating that part of the incident *x*-polarized wave transmits to *y*-polarized wave. This peak arises from the combination of the chiroptical response of the metamolecule and the FP resonance of the cavity.

The measured linear transmission coefficients of chiral metamolecules are shown in Figure 3(c), where the *t*_*xx*_ overlaps with *t*_*yy*_ reaching 80% while the *t*_*yx*_ is almost the same as *t*_*xy*_ with a low value of 2%. The measurement results match the numerical simulation data very well (Figure 3(a)). Based on the analysis above, the chiral response can be improved significantly by using FP-like cavity. In Figure 3(d), we present the measurement of the linear transmission coefficients of the FP cavity embedded with the chiral metamolecules. The *t*_*yx*_ and *t*_*xy*_ exhibit a peak at 12.6 GHz with the magnitudes of 40% and 5.5%, respectively, leading to a significant difference in the cross-polarization transmission coefficients, that in turn, improves the OA of the pure chiral resonator pronouncedly. Nevertheless, *t*_*xx*_ and *t*_*yy*_ are almost the same with each other, and both of them peak at 12.6 GHz with a transmission of 34%. The experimental data are in a good agreement with simulations (Figure 3(b)), except for the minor deviations in the transmission magnitudes. It is attributed to the slight discrepancies in the geometrical parameters between the modeling and fabrication and the finite dimensions of the fabricated sample but not the simulated one [51]. The difference between experiment and theory is also caused by the cross-polarization of the paired horn antennas [56], the measurement uncertainty, and assembly error [57]. The finesse of the FP cavity formed by two metamirrors without chiral medium is ~68 [58], shown in [Supplementary-material supplementary-material-1] from the supplementary information (SI).

The chirality enhancement results from the fact that the spin of the wave is not flipped by the reflectors, leading to constructive chiral interaction of the wave with the chiral molecule placed inside the FP cavity. To better characterize the chiral enhancement of the cavity, four transmission coefficients, *t*_−−_, *t*_++_, *t*_−+_, and *t*_+−_ representing left-to-left, right-to-right, right-to-left, and left-to-right polarized transmission conversion efficiencies, respectively [59–61], are calculated from the linear transmission coefficients via the equation

(1) $$\begin{array}{c} t_{\text{circ}}=\left(\begin{array}{cc} t_{++} & t_{+-}\\ t_{-+} & t_{--} \end{array}\right)=\frac{1}{2}\left(\begin{array}{cc} t_{xx}+t_{yy}+i\left(t_{xy}-t_{yx}\right) & t_{xx}-t_{yy}-i\left(t_{xy}+t_{yx}\right)\\ t_{xx}-t_{yy}+i\left(t_{xy}+t_{yx}\right) & t_{xx}+t_{yy}-i\left(t_{xy}-t_{yx}\right) \end{array}\right). \end{array}$$

For a pure chiral resonator, the rotation angle *ϕ* is proportional to its chirality parameter *κ*, which is expressed as

**Figure 4:**
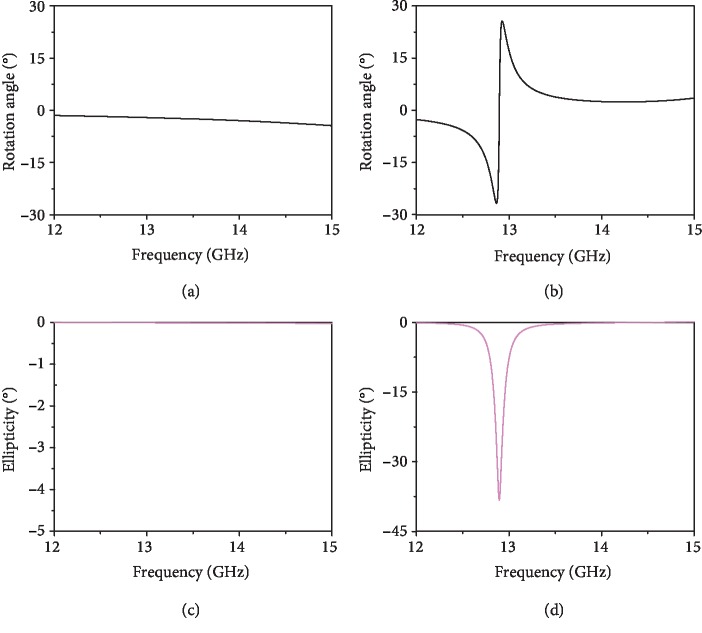
(a, b) The CST-simulated rotation angle *ϕ* of (a) pure chiral metamolecule and (b) FP cavity configuration and (c, d) ellipticity *η* of (c) pure chiral metamolecule and (d) FP cavity configuration structures with *θ*_1_ = 45° and *θ*_2_ = 60°.

(2) ϕ=Reκk0d=12argt++−argt−−,

where *k*_0_ is the wave vector in vacuum and *d* is the thickness of the chiral metamaterial. Therefore, the rotation angle *ϕ* measures the strength of optical activity (OA). As shown in Figure 4(a), the rotation angle of the pure chiral resonator (i.e., bilayer twisted cross wires) is *ϕ* = −2° at *f* = 12.9 GHz, and it is increased to *ϕ* = −26.7° inside the cavity (Figure 4(b)). Thus, our proposed structure can significantly improve the chirality of the pure chiral molecule. In Figure 4(c) and 4(d), we simulate the ellipticity *η* = arctan((|*t*_++_|−|*t*_−−_|)/(|*t*_++_|+|*t*_−−_|))for both structures. At the resonant frequency of *f* = 12.9 GHz, the ellipticity reaches its highest value of *η* = −38.3° with the cavity configuration (Figure 4(d)), while *η* is equal to 0° for the pure chiral metamolecule in the spectral region from 12 to 15 GHz (Figure 4(c)). The above numerical simulation shows that the FP cavity can dramatically enhance the detection sensitivity of the chirality of the metamolecules in both the forms of OA and circular dichroism (CD). Moreover, in [Supplementary-material supplementary-material-1] from SI, we numerically show that both *ϕ* and *η* can be flipped by reversing the chirality of the metamolecule by setting *θ*_1_ = 60° and *θ*_2_ = 45° while maintaining the other geometrical parameters.

**Figure 5:**
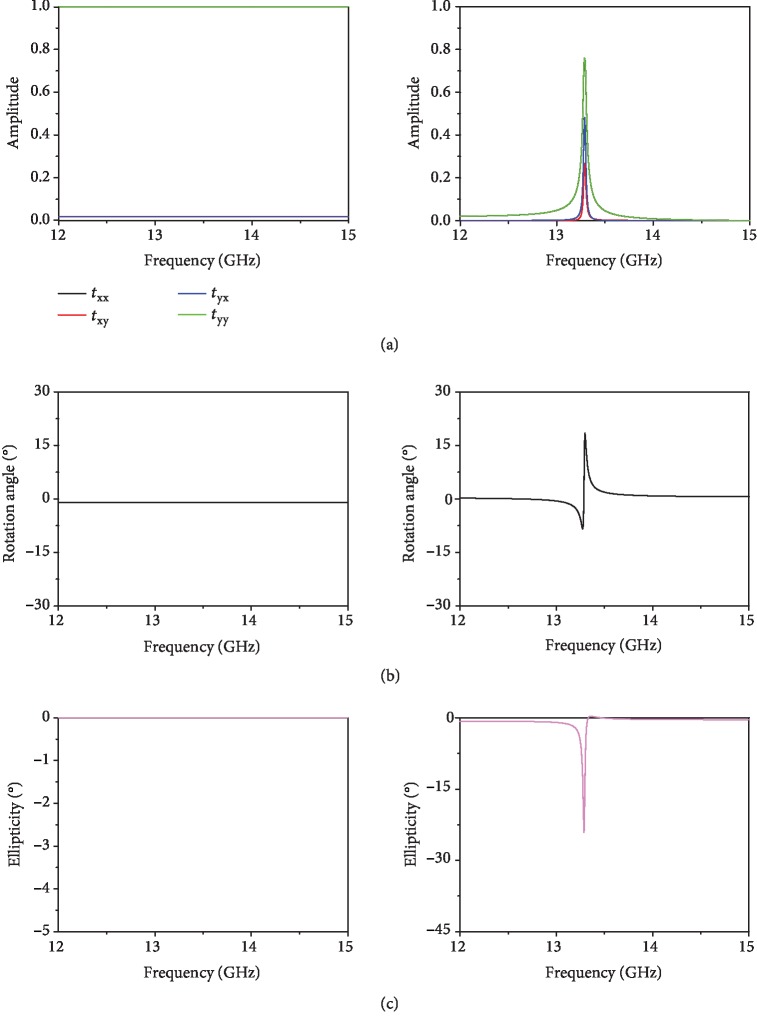
The theoretically calculated (a) linear transmission coefficients *t*_*yx*_, *t*_*xy*_, *t*_*xx*_, and *t*_*yy*_, (b) rotation angle *ϕ*, and (c) ellipticity *η* of the pure chiral medium (left column) and FP cavity embedded by the chiral medium (right column) structures. The rotation angle of the pure chiral medium is defined by *ϕ* = −1°.

To prove the above numerical finding, we derive a theoretical model of FP cavity filled with a chiral medium with a linearly polarized incidence. We have performed detailed theoretical analyses to elucidate the interaction between the light and chiral medium inside the FP cavity, where the complex reflection and transmission coefficients are derived (Note 3 of SI). In Figure 5, we have presented the theoretical calculations of the linear transmission coefficients, polarization rotation angle, and ellipticity of both pure chiral medium and FP cavity filled with the chiral medium. As is seen, the theoretical calculation qualitatively reproduced the CST numerical simulation and experimental measurement. From this study, we theoretically conclude that the chiroptical responses of the chiral molecule placed inside the FP cavity can be significantly enhanced using the multiple reflections with preserved spin. In note 4 of SI, we have further studied the fundamental mechanism of linearly polarized wave-chiral resonator interaction. Note that the *ϕ* of the chiral molecule cannot be significantly improved inside a FP cavity formed by a pair of parallel gratings (see note 5 of SI).

In note 6 of SI, we further show that the metamirror preserves the helicity, in contrast to a normal metallic mirror. As is seen, the HPM mirror enables the reflected waves to maintain their handednesses ([Supplementary-material supplementary-material-1](a)); however, in a normal metallic mirror, this reflection changes RCP to LCP wave and vice versa ([Supplementary-material supplementary-material-1](b)), which thus damaging any beneficial influence of the cavity on the optical activity enhancement. Moreover, in a conventional FP cavity formed by a pair of Cu films ([Supplementary-material supplementary-material-1](a)), each reflection flips the handedness of the wave and the chiral responses of the forward propagating wave are canceled by the backward propagating wave inside the FP cavity, leading to a zero optical activity ([Supplementary-material supplementary-material-1](b)).

A key feature that distinguishes our proposed cavity from the previous structures that provide localised EM fields for enhancing the chiral signals is that the large enhancement does not only occur at the near field around the resonators. We now study the chirality enhancement as a function of location (*l*_1_) inside the FP cavity ([Supplementary-material supplementary-material-1](a)). In [Supplementary-material supplementary-material-1](b), we numerically demonstrate the *ϕ* spectra at five different positions of *l*_1_ = 3.25, 4.25, 5.25, 6.25, and 7.25 mm inside the cavity. We find that the chirality (*ϕ*) enhancement increases as it is moving away from the grating towards the center of FP-like cavity, which is outside the near-field areas around the grating. The FP cavity alone formed by the two handedness-preserving metamirrors does not exhibit any chiroptical effect due to the mirror symmetry of the system. The chirality here is only introduced by inserting a layer of chiral metamolecule inside the cavity, which serves as the origin of the chiroptical response in the combined system. In other words, the FP cavity does not create the chiroptical effect but enhances it.

## 3. Discussion

In summary, our simulations and measurements show that the FP cavity constructed by a pair of metasurface mirrors can improve the optical activity of the chiral molecule placing inside the FP cavity. Moreover, our method can readily extend to the other frequency regions, simply by varying the size of the structure. Nevertheless, metal losses and fabrication challenges may be problematic when approaching visible regions. For decades, the FP cavity has provided a wide range of applications such as laser resonators, optical wavemeters, dichroic filters, and add-drop multiplexers. However, FP cavity made of conventional mirrors cannot be used for enhancing the chiral sensing capability due to the reversal of spin of light upon each reflection. In this work, our proposed FP-like cavity system based on spin-reserving reflectors can advance the technique of enhancement of optical activity. Note that owing to the scaling characteristic of Maxwell's equations, our proposed scheme can be straightforward extended to other higher frequencies, such as visible-infrared and terahertz regions, with a scaledown of the proposed FP cavity, and open up a novel possibility of chiroptical spectroscopy.

## 4. Materials and Methods

### 4.1. Experiment

During the experiment, the two standard gain horn antennas are placed symmetrically on both sides of the measured samples and the aperture center of the horn antenna is aligned along the central axis of the samples to concentrate the EM waves on the samples. Meanwhile, the samples are surrounded by the tapered broadband absorbing material to reduce the diffraction of EM waves. The two standard gain horn antennas are, respectively, connected to the two ports of the vector network analyzer, one of the horn antennas is used as the transmitter to convert the EM signals into specified-polarized spatial EM waves and the EM waves are irradiated into the samples after propagating a certain distance in free space. After penetrating the samples, the EM waves are transmitted in free space for a certain distance and then received by another horn antenna. By changing the polarization direction of the receiving horn antenna, EM waves of different polarization states can be received and converted into EM signals for postprocessing by the vector network analyzer.

## Supplementary Materials

Supplementary Materials Note 1: the finesse of FP cavity without chiral medium. Note 2: the CST simulation of reversal chiral metamolecules in the FP cavity. Note 3: physical model of Fabry–Pérot-like cavity. Note 4: mechanism of linearly polarized wave-chiral resonator interaction. Note 5: the CST simulation of chiral meta-atom in the FP cavity formed by the paired parallel gratings. Note 6: the CST simulation of reflection spectra of handedness-preserving metasurfaces (HPM) and Cu film. Note 7: the CST simulation of chiral meta-atom in the FP cavity formed by a pair of Cu films. Note 8: chirality enhancement as a function of location inside the FP cavity. Figure S1: (a) design schematics of the FP cavity formed by two metamirrors without chiral medium. (b) The CST-simulated linear transmission coefficients *t*_*yx*_, *t*_*xy*_, *t*_*xx*_, and *t*_*yy*_ for the FP cavity, where *v*_0_ = 13.785 GHz is the center frequency of the resonant peak and Δ*v* = 0.183 GHz the linewidth of the resonance (full-width half maximum). Figure S2: the CST-simulated (a, b) rotation angle of (a) pure chiral meta-atom and (b) FP cavity configuration and (c, d) ellipticity *η* of (c) pure chiral meta-atom and (d) FP cavity configuration structures with *θ*_1_ = 60° and *θ*_2_ = 45°. Figure S3: the scheme of the metal grid layer. The black, blue, and green arrows represent the incident, reflected, and transmitted electromagnetic (EM) waves. The pink arrows represent the emissivity of the metal grid layer. Figure S4: schematic of air/metal/air trilayered structure in which the middle grating is treated as a homogeneous metal layer with a refractive index of *n*_*m*_.. Figure S5: schematic of the metal/air/metal trilayered reflector in which the top grating is treated as a homogeneous metal layer with a refractive index of *n*_*m*_.. Figure S6: schematic of multiple reflections and transmissions in the FP-like cavity. Figure S7; schematic of multiple reflection and transmission in the FP-like cavity with the chiral medium. Figure S8: the enhancement mechanism of the FP resonance in (a) dual-layered AB structure, (b) trilayered ABC structure, and (c) multilayered ABCDE structure (green and red arrows represent *y*- and *x*-polarized wave, respectively). Figure S9: (a) design schematic and (b) CST-simulated rotation angle of a chiral molecule (*θ*_1_ = 45° and *θ*_2_ = 60°) placed inside a FP cavity formed by two parallel gratings. Figure S10: (a) a HPM mirror at a vertical incidence reflects the RCP and LCP waves and preserves their handedness (top panel). Reflection spectra of the co- and cross-polarized components of the HPM (bottom panel). (b) A normal metallic mirror under a vertical incidence reverses the handedness of both RCP and LCP waves in reflection (top panel). Reflection spectra of the co- and cross-polarized components of the normal metallic mirror (bottom panel). Figure S11: (a) design schematics and (b) CST-simulated rotation angle of the chiral element (*θ*_1_ = 45° and *θ*_2_ = 60°) placed inside a conventional FP cavity formed by a pair of Cu films. Figure S12: (a) design schematics of the chiral molecule placed at the various positions inside a FP cavity formed by two metamirrors. (b) The CST-simulated rotation angle (*ϕ*) spectra at five different positions of *l*_1_ = 3.25, 4.25, 5.25, 6.25, and 7.25 mm inside the cavity.
