# Supplementary material for: Chirality Enhancement Using Fabry–Pérot-Like Cavity
Source: Research (Wash D C). 2020 Feb 28;2020:7873581. doi: 10.34133/2020/7873581 (PMC7064819; doi:10.34133/2020/7873581)
Supplement: Supplementary Materials — Note 1: the finesse of FP cavity without chiral medium. Note 2: the CST simulation of reversal chiral metamolecules in the FP cavity. Note 3: physical model of Fabry–Pérot-like cavity. Note 4: mechanism of linearly polarized wave-chiral resonator interaction. Note 5: the CST simulation of chiral meta-atom in the FP cavity formed by the paired parallel gratings. Note 6: the CST simulation of reflection spectra of handedness-preserving metasurfaces (HPM) and Cu film. Note 7: the CST simulation of chiral meta-atom in the FP cavity formed by a pair of Cu films. Note 8: chirality enhancement as a function of location inside the FP cavity. Figure S1: (a) design schematics of the FP cavity formed by two metamirrors without chiral medium. (b) The CST-simulated linear transmission coefficients tyx, txy, txx, and tyy for the FP cavity, where v0 = 13.785 GHz is the center frequency of the resonant peak and Δv = 0.183 GHz the linewidth of the resonance (full-width half maximum). Figure S2: the CST-simulated (a, b) rotation angle of (a) pure chiral meta-atom and (b) FP cavity configuration and (c, d) ellipticity η of (c) pure chiral meta-atom and (d) FP cavity configuration structures with θ1 = 60° and θ2 = 45°. Figure S3: the scheme of the metal grid layer. The black, blue, and green arrows represent the incident, reflected, and transmitted electromagnetic (EM) waves. The pink arrows represent the emissivity of the metal grid layer. Figure S4: schematic of air/metal/air trilayered structure in which the middle grating is treated as a homogeneous metal layer with a refractive index of nm.. Figure S5: schematic of the metal/air/metal trilayered reflector in which the top grating is treated as a homogeneous metal layer with a refractive index of nm.. Figure S6: schematic of multiple reflections and transmissions in the FP-like cavity. Figure S7; schematic of multiple reflection and transmission in the FP-like cavity with the chiral medium. Figure S8: the enhancement mechanism of t [file 7873581.f1.docx]

**Supplementary Materials for**

**Chirality Enhancement using Fabry–Pérot-like Cavity**

Jia Xin Bao^1,†^, Ning Liu^1^, Han Wei Tian^3^, Qiang Wang^3^, Tie Jun Cui^3^, Wei Xiang Jiang^3^, ^*^, Shuang Zhang^2^, ^*^, and Tun Cao^1, †*^

^1^ School of Optoelectronic Engineering and Instrumentation Science, Dalian University of Technology, Dalian 116024, China.

^2^ School of Physics & Astronomy, university of Birmingham, Birmingham, B15 2TT, UK

^3^State Key Laboratory of Millimeter Waves, School of Information Science and Engineering, Southeast University, Nanjing 210096, China

^†^These authors contributed equally to this work.

^*^Corresponding authors: W. X. Jiang: [wxjiang81@seu.edu.cn](mailto:wxjiang81@seu.edu.cn), T. Cao: [caotun1806@dlut.edu.cn](mailto:caotun1806@dlut.edu.cn) S. Zhang: [S.Zhang@bham.ac.uk](mailto:S.Zhang@bham.ac.uk)

**This PDF file includes:**

Supplementary Text Note.1 to Note 8

Supplementary Figures S1 to S12

**Note. 1 The finesse of FP cavity without chiral medium.**

Figure S1(a) schematically shows the Fabry-Perot (FP) cavity formed by two metamirrors. In Figure S1(b), we present the CST simulated transmission coefficients of $t_{xx}$, $t_{yx}$, $t_{yy}$and $t_{xy}$for the FP cavity without chiral medium as shown in Figure R1(a), where $t_{xx}$,$t_{yx}$, $t_{yy}$and $t_{xy}$ correspond to *x*(input)-to-*x* (output), *x* (input)-to-*y* (output), *y* (input)-to-*y* (output), and *y* (input)-to-*x* (output) polarised transmission conversion efficiencies.

**Figure S1**. (a) Design schematics of the FP cavity formed by two metamirrors without chiral medium. (b) The CST simulated linear transmission coefficients $t_{yx}$, $t_{xy}$,$t_{xx}$and $t_{yy}$ for the FP cavity, where $\nu_{0}$=13.785 GHz is the center frequency of the resonant peak and $\Delta\nu$= 0.183 GHz the linewidth of the resonance (full width half maximum).

The condition for constructive interference within the cavity, and therefore resonant behavior, is $2nL=m\lambda$.$n$refractive index, $L$the length of the cavity, $\lambda$the center wavelength of the resonant peak, and m=1,2,3... We can rewrite expression for the resonant frequencies of the FP cavity as$\nu=\frac{mc}{2nL}=\frac{mc}{2L}$, where *c* is the light speed in vacuum. Therefore, the frequency difference between resonances is $c/2L$ so called the free spectral range. The finesse is defined as the free spectral range divided by the linewidth of the resonance [52]:

$F=\frac{c/\left(2L\right)}{\Delta\nu}=\frac{3\times10^{8}/\text{（}2\times12\times10^{-3}\text{）}}{13.875\times10^{9}-13.692\times10^{9}}=68.31$.

**Note. 2 The CST simulation of reversal chiral meta-atom in the FP cavity.**

**Figure S2**. The CST simulated (a, b) rotation angle of (a)pure chiral meta-atom, (b)FP cavity configuration, and (c, d) ellipticity *η* of (c)pure chiral meta-atom, (d)FP cavity configuration structures with *θ_1_* = 60° and *θ_2_* = 45°.

**Note. 3 Physical model of Fabry-P****érot-like cavity**

A physical model was employed to explain the enhancement of optical activity (OA) of the chiral element using the Fabry-Pérot (FP)-like cavity, where the complex reflection and transmission coefficients of the FP-like cavity are theoretically calculated.

**Note 3.1. The metal grid layer**

**Figure S3.** The scheme of the metal grid layer. The black, blue, and green arrows represent the incident, reflected, and transmitted electromagnetic (EM) waves. The pink arrows represent the emissivity of the metal grid layer.

Herein, the metal grid layer acts as an antennae array. As seen in Fig.S3, when the metal grid layer was normally illuminated by an electromagnetic (EM) wave (indicated by a black arrow), a part of the incident EM wave can directly transmit through the apertures without losses (shown by a cyan arrow) and the other part of the incident EM wave can impinge the antenna to launch the EM waves towards both +z and –z-directions (shown by pink arrows). By treating the emissivity along -z-direction as the reflection coefficient (indicated by a blue arrow) [1], we can obtain

$t_{1}=1+e_{1}$ (S1)

$\left|t_{1}\right|^{2}+\left|r_{1}\right|^{2}=1$ (S2)

where$r_{1}$,$t_{1}$,$e_{1}$are the complex reflection, transmission and emission coefficients, respectively, and $\left|r_{1}\right|=\left|e_{1}\right|$.

Assuming that$r_{1}=\left|r_{1}\right|e^{i\beta}$, we can get $r_{1}$and $t_{1}$ for the EM wave transmitting from air to the metal grid layer.

$r_{1}=-\cos\beta\cdot e^{i\beta}$ (S3)

$t_{1}=1-\cos\beta\cdot e^{i\beta}$ (S4)

**Note 3.2. The metal/air/metal tri-layered mirror reflector**

Firstly, we derive the complex transmission and reflection coefficients through the grating monolayer residing at the interface between two air media. The middle ultrathin grating is treated as a homogeneous metal layer with a refractive index nm and thickness dm. The refractive index of the air layer is n_1_=n_3_=1. For the tri-layered structure shown in Fig. S4, the Fresnel equations [2] can be expressed by,

$t_{2}^{-1}=\cos(n_{m}k_{0}d_{m})-\frac{i}{2}(\frac{1}{n_{m}}+n_{m})\sin(n_{m}k_{0}d_{m})$ (S5)

$r_{2}t_{2}^{-1}=-\frac{i}{2}(\frac{1}{n_{m}}-n_{m})\sin(n_{m}k_{0}d_{m})$ (S6)

**Figure S4.** Schematic of air/metal/air tri-layered structure in which the middle grating is treated as a homogeneous metal layer with a refractive index of n_m_. The black, blue, and green arrows represent the incident, reflected, and transmitted EM waves.

We then relate the susceptibility of metal medium to the polarisability of an individual antenna as,

$\chi_{e}=\frac{\alpha_{e}}{L^{2}d_{m}}$ (S7)

where$\alpha_{e}$is the antenna polarizability, $L$ is the in-plane lattice constant, and $d_{m}$ is the thickness of grating. Considering the localised surface plasmon resonance (LSPR) of the antenna, the antenna polarisability can be expressed by a Lorentzian function [3]

$\alpha_{e}\propto-\frac{1}{\omega-\omega_{0}+i\gamma}$ (S8)

where $\omega$ is frequency, $\omega_{0}$is oscillator frequency and$\gamma$ is damping parameter. Given by the metal permittivity of$\varepsilon=1+\chi_{e}\approx\chi_{e}=\frac{\alpha_{e}}{L^{2}d_{m}}$, the refractive index of the metal medium can be expressed as,

$n_{m}\approx\frac{1}{L}\sqrt{\frac{\alpha_{e}}{d_{m}}}$ (S9)

With an ultrathin dm ~0，we have

$t_{2}^{-1}=1-i\omega P$ (S10)

$r_{2}t_{2}^{-1}=i\omega P$ (S11)

$P=\frac{\alpha_{e}}{2cL^{2}}=-\frac{g}{\omega-\omega_{0}+i\gamma}$ (S12)

where g is a parameter that indicates the coupling strength between the antenna and incident light.

Next, we study the configuration of the entire mirror reflector consisting of a metal-air-metal tri-layered structure, where the top metal grating and bottom metal grid layer is spaced by an air interlayer, as shown in Fig. S5.

**Figure S5.** Schematic of metal/air/metal tri-layered reflector in which the top grating is treated as a homogeneous metal layer with a refractive index of$n_{m}$. The black, blue, and green arrows represent the incident, reflected, and transmitted EM waves.

The transmission coefficient of the tri-layered reflector mirror can be expressed using Eq. (S13),

$t_{m}=t_{1}t_{2}e^{i\frac{\alpha}{2}}+t_{1}e^{i\frac{\alpha}{2}}r_{2}e^{i\frac{\alpha}{2}}r_{1}e^{i\frac{\alpha}{2}}t_{2}+......=\frac{t_{1}t_{2}e^{i\frac{\alpha}{2}}}{1-r_{1}r_{2}e^{i\alpha}}$ (S13)

where $\alpha=2n_{3}k_{0}d_{3}$ is the round trip phase of the air layer, d_3_ the thickness of the air, n_3_=1 the refractive index of the air layer, and k0 the wave vector in the air. By placing equation (S10)-(S12) into equation (S13), we can obtain

$t_{m}=\frac{t_{1}t_{2}e^{i\frac{\alpha}{2}}}{1-r_{1}r_{2}e^{i\alpha}}=\frac{t_{1}e^{i\frac{\alpha}{2}}}{\frac{1}{t_{2}}-r_{1}\frac{r_{2}}{t_{2}}e^{i\alpha}}=\frac{t_{1}e^{i\frac{\alpha}{2}}}{1+\frac{(1+r_{1}e^{i\alpha})i\omega g}{\omega-\omega_{0}+i\gamma}}$ (S14)

We will then study the effect of the polarization of the incident electric (E) field on the transmission coefficients. When the incident E-field polarises along the long axis of the grating with$\omega g\gg\gamma$ [3], the transmission coefficient can be expressed by

$t_{m}=\frac{t_{1}e^{i\frac{\alpha}{2}}}{1+\frac{(1+r_{1}e^{i\alpha})i\omega g}{\omega-\omega_{0}+i\gamma}}=\frac{\left(\omega-\omega_{0}\right)t_{1}e^{i\frac{\alpha}{2}}}{\left(\omega-\omega_{0}\right)+(1+r_{1}e^{i\alpha})i\omega g}$ (S15)

whilst for the incident E-field polarising along the short axis with $g=0$ [3], the transmission coefficient is shown as,

$t_{m}=\frac{t_{1}e^{i\frac{\alpha}{2}}}{1+\frac{(1+r_{1}e^{i\alpha})i\omega g}{\omega-\omega_{0}+i\gamma}}=t_{1}e^{i\frac{\alpha}{2}}$ (S16)

Analogously, the reflection coefficient can be expressed as,

$r_{m}=r_{2}+t_{2}e^{i\frac{\alpha}{2}}r_{1}e^{i\frac{\alpha}{2}}t_{2}+t_{2}e^{i\frac{\alpha}{2}}r_{1}e^{i\frac{\alpha}{2}}t_{2}r_{2}e^{i\frac{\alpha}{2}}r_{1}e^{i\frac{\alpha}{2}}t_{2}+......=r_{2}+\frac{t_{2}t_{2}r_{1}e^{i\alpha}}{1-r_{1}r_{2}e^{i\alpha}}$ (S17)

By placing equation (S10)-(S12) into equation (S17), we can obtain

$r_{m}=r_{2}+\frac{t_{2}t_{2}r_{1}e^{i\alpha}}{1-r_{1}r_{2}e^{i\alpha}}=\frac{\left(-i\omega g\right)\left(\omega-\omega_{0}+i\gamma+\left(r_{1}e^{i\alpha}+1\right)i\omega g\right)+r_{1}e^{i\alpha}\left(\omega-\omega_{0}+i\gamma\right)^{2}}{\left(\omega-\omega_{0}+i\gamma+i\omega g\right)\left(\omega-\omega_{0}+i\gamma+\left(r_{1}e^{i\alpha}+1\right)i\omega g\right)}$ (S18)

The reflection coefficient is also dependent on the polarisation of the incident E-field. For the incident E-field that polarises along the long axis of the grating with$\omega g\gg\gamma$, the reflection coefficient can be expressed by

$r_{m}=\frac{\left(r_{1}e^{i\alpha}+1\right)\omega^{2}g^{2}-\left(\omega-\omega_{0}\right)i\omega g+r_{1}e^{i\alpha}\left(\omega-\omega_{0}\right)^{2}}{\left(\omega-\omega_{0}+i\omega g\right)\left(\omega-\omega_{0}+\left(r_{1}e^{i\alpha}+1\right)i\omega g\right)}$ (S19)

For the incident E-field polarising along the short axis of the grating with$g=0$, the transmission coefficient is expressed by,

$r_{m}=\frac{r_{1}e^{i\alpha}\left(\omega-\omega_{0}+i\gamma\right)^{2}}{\left(\omega-\omega_{0}+i\gamma\right)\left(\omega-\omega_{0}+i\gamma\right)}\approx r_{1}e^{i\alpha}$ (S20)

**Note 3.3.The FP-like cavity**

**Figure S6.** Schematic of multiple reflections and transmissions in the FP-like cavity.

As seen in Fig. S6, the FP-like cavity is composed by a pair of orthogonal grating resonators that are printed on the F4B dielectric layer integrated with a ground Cu grid array. The $t_{ij}$ represents the complex linear transmission matrix that connects the transmitted E-field vector $E_{i}$ and the input E-field vector $E_{j}$ by $E_{i}=t_{ij}\times E_{j}$, where ‘i’ and ‘j’ are symbols for plane waves linearly polarising along the x- and y- axes [4]. The $r_{ij}$ represents the complex linear reflection matrix that connects the reflected E-field vector $E_{i}$ and the input E-field vector $E_{j}$ by $E_{i}=r_{ij}\times E_{j}$, where ‘i’ and ‘j’ are symbols for plane waves linearly polarising along the x- and y-axes. Herein, for an incident plane wave linearly polarizing along the x-axis, one can obtain the linear transmission coefficients of $t_{xx}$and $t_{yx}$ using x- and y- polarized receiver antenna respectively. Likewise, $t_{yy}$and $t_{xy}$ can also be obtained by launching the y-polarized incident wave. Note that, the complex transmission coefficients of circularly polarised light can be obtained from the linear transmission coefficients [5].

The Jones matrix is used to characterize the mirror reflectors and defined as [4]:

$t=\left[\begin{matrix} t_{xx} & t_{xy} \\ t_{yx} & t_{yy} \end{matrix}\right]$ (S21)

$r=\left[\begin{matrix} r_{xx} & r_{xy} \\ r_{yx} & r_{yy} \end{matrix}\right]$ (S22)

By placing Eq. (S15-S16) into Eq. (S21) and Eq. (S19-S20) into Eq. (22), the Jones matrices of the two orthogonal mirror reflector can be expressed by

$t_{m1}=\left[\begin{matrix} t_{xx} & t_{xy} \\ t_{yx} & t_{yy} \end{matrix}\right]=\left[\begin{matrix} \frac{\left(\omega-\omega_{0}\right)t_{1}e^{i\frac{\alpha}{2}}}{\left(\omega-\omega_{0}\right)+(1+r_{1}e^{i\alpha})i\omega g} & 0 \\ 0 & t_{1}e^{i\frac{\alpha}{2}} \end{matrix}\right]$ (S23)

$r_{m1}=\left[\begin{matrix} r_{xx} & r_{xy} \\ r_{yx} & r_{yy} \end{matrix}\right]=\left[\begin{matrix} \frac{\left(r_{1}e^{i\alpha}+1\right)\omega^{2}g^{2}-\left(\omega-\omega_{0}\right)i\omega g+r_{1}e^{i\alpha}\left(\omega-\omega_{0}\right)^{2}}{\left(\omega-\omega_{0}+i\omega g\right)\left(\omega-\omega_{0}+\left(r_{1}e^{i\alpha}+1\right)i\omega g\right)} & 0 \\ 0 & r_{1}e^{i\alpha} \end{matrix}\right]$ (S24)$t_{m2}=\left[\begin{matrix} t_{xx} & t_{xy} \\ t_{yx} & t_{yy} \end{matrix}\right]=\left[\begin{matrix} t_{1}e^{i\frac{\alpha}{2}} & 0 \\ 0 & \frac{\left(\omega-\omega_{0}\right)t_{1}e^{i\frac{\alpha}{2}}}{\left(\omega-\omega_{0}\right)+(1+r_{1}e^{i\alpha})i\omega g} \end{matrix}\right]$ (S25)

$r_{m2}=\left[\begin{matrix} r_{xx} & r_{xy} \\ r_{yx} & r_{yy} \end{matrix}\right]=\left[\begin{matrix} r_{1}e^{i\alpha} & 0 \\ 0 & \frac{\left(r_{1}e^{i\alpha}+1\right)\omega^{2}g^{2}-\left(\omega-\omega_{0}\right)i\omega g+r_{1}e^{i\alpha}\left(\omega-\omega_{0}\right)^{2}}{\left(\omega-\omega_{0}+i\omega g\right)\left(\omega-\omega_{0}+\left(r_{1}e^{i\alpha}+1\right)i\omega g\right)} \end{matrix}\right]$ (S26)

We define

$X=\frac{\left(\omega-\omega_{0}\right)}{\left(\omega-\omega_{0}\right)+(1+r_{1}e^{i\alpha})i\omega g}$ (S27) $Y=\frac{\left(r_{1}e^{i\alpha}+1\right)\omega^{2}g^{2}-\left(\omega-\omega_{0}\right)i\omega g+r_{1}e^{i\alpha}\left(\omega-\omega_{0}\right)^{2}}{\left(\omega-\omega_{0}+i\omega g\right)\left(\omega-\omega_{0}+\left(r_{1}e^{i\alpha}+1\right)i\omega g\right)}$ (S28)

So the Jones matrices can be simplified as

$t_{m1}=\left[\begin{matrix} t_{xx} & t_{xy} \\ t_{yx} & t_{yy} \end{matrix}\right]=\left[\begin{matrix} t_{1}e^{i\frac{\alpha}{2}}X & 0 \\ 0 & t_{1}e^{i\frac{\alpha}{2}} \end{matrix}\right]$ (S29)

$r_{m1}=\left[\begin{matrix} r_{xx} & r_{xy} \\ r_{yx} & r_{yy} \end{matrix}\right]=\left[\begin{matrix} Y & 0 \\ 0 & r_{1}e^{i\alpha} \end{matrix}\right]$ (S30)

$t_{m2}=\left[\begin{matrix} t_{xx} & t_{xy} \\ t_{yx} & t_{yy} \end{matrix}\right]=\left[\begin{matrix} t_{1}e^{i\frac{\alpha}{2}} & 0 \\ 0 & t_{1}e^{i\frac{\alpha}{2}}X \end{matrix}\right]$ (S31)

$r_{m2}=\left[\begin{matrix} r_{xx} & r_{xy} \\ r_{yx} & r_{yy} \end{matrix}\right]=\left[\begin{matrix} r_{1}e^{i\alpha} & 0 \\ 0 & Y \end{matrix}\right]$ (S32)

The transmission coefficient of the whole FP-like cavity model can be written as

$\begin{matrix} t_{FP}=t^{\left(1\right)}+t^{\left(2\right)}+t^{\left(3\right)}+... \\ =t_{m2}\left(t_{m1}e^{in_{1}k_{0}l}\right)+t_{m2}\left(r_{m1}r_{m2}e^{i2n_{1}k_{0}l}\right)\left(t_{m1}e^{in_{1}k_{0}l}\right)+t_{m2}\left(r_{m1}r_{m2}e^{i2n_{1}k_{0}l}\right)\left(r_{m1}r_{m2}e^{i2n_{1}k_{0}l}\right)\left(t_{m1}e^{in_{1}k_{0}l}\right)+...... \end{matrix}$ (S33)

Here，l is the length of the FP-like cavity

$r_{m1}r_{m2}=\left[\begin{matrix} r_{1}e^{i\alpha} & 0 \\ 0 & Y \end{matrix}\right]\left[\begin{matrix} Y & 0 \\ 0 & r_{1}e^{i\alpha} \end{matrix}\right]=\left[\begin{matrix} Yr_{1}e^{i\alpha} & 0 \\ 0 & Yr_{1}e^{i\alpha} \end{matrix}\right]$ (S34)

By placing eq. (S29), eq. (S30) and eq. (S34) into eq. (S33), the transmission coefficient of the FP-like cavity model can be rewritten as

$t_{FP}=\frac{e^{in_{1}k_{0}l}}{1-2Yr_{1}e^{i\alpha}e^{i2n_{1}k_{0}l}+r_{1}^{2}e^{2i\alpha}Y^{2}e^{i2n_{1}k_{0}l}}\left[\begin{matrix} t_{1}^{2}e^{i\alpha}X\left(1-Yr_{1}e^{i\alpha}e^{i2n_{1}k_{0}l}\right) & 0 \\ 0 & t_{1}^{2}e^{i\alpha}X\left(1-Yr_{1}e^{i\alpha}e^{i2n_{1}k_{0}l}\right) \end{matrix}\right]$ (S35)

where$X=\frac{\left(\omega-\omega_{0}\right)}{\left(\omega-\omega_{0}\right)+(1+r_{1}e^{i\alpha})i\omega g}$,$Y=\frac{\left(r_{1}e^{i\alpha}+1\right)\omega^{2}g^{2}-\left(\omega-\omega_{0}\right)i\omega g+r_{1}e^{i\alpha}\left(\omega-\omega_{0}\right)^{2}}{\left(\omega-\omega_{0}+i\omega g\right)\left(\omega-\omega_{0}+\left(r_{1}e^{i\alpha}+1\right)i\omega g\right)}$, $\omega$ the frequency of the wave, $\omega_{0}$ the oscillator frequency of individual antenna, $\alpha$ the round trip phase of the air layer, l the length of the cavity, n1=1 the refractive index of the air layer, k0 the wave vector in the air, g indicates the coupling strength between the antenna and incident light, $r_{1}$and $t_{1}$the complex reflection and transmission of the metal grid layer respectively.

**Note 3.4. The FP-like cavity with chiral medium**

**Figure S7.** Schematic of multiple reflection and transmission in the FP-like cavity with the chiral medium.

We use a rotation matrix to simplify the Jones matrix of chiral medium,

(S36)

The transmission coefficient of the whole FP-like cavity model can be written as,

$\begin{matrix} t_{FP}=t^{\left(1\right)}+t^{\left(2\right)}+t^{\left(3\right)}+... \\ =t_{m2}\left(t_{c}t_{m1}e^{in_{1}k_{0}l}\right)+t_{m2}\left(t_{c}r_{m1}t_{c}r_{m2}e^{i2n_{1}k_{0}l}\right)\left(t_{c}t_{m1}e^{in_{1}k_{0}l}\right)+t_{m2}\left(t_{c}r_{m1}t_{c}r_{m2}e^{i2n_{1}k_{0}l}\right)\left(t_{c}r_{m1}t_{c}r_{m2}e^{i2n_{1}k_{0}l}\right)\left(t_{c}t_{m1}e^{in_{1}k_{0}l}\right)+... \end{matrix}$ (S37)

By placing eq. (S29)-(S32) and eq. (S36) into eq. (S37), the transmission coefficient of the FP-like cavity model can be rewritten as

(S38)

Here，

(S39) (S40)

(S41)

(S42)

(S43)

To prove how our FP-like cavity can enhance the chirality，we will calculate the amplitude and phase of transmission coefficients, rotation angle, and ellipticity of the pure chiral medium and the chiral medium that is placed inside the FP-like cavity. The chiral medium is defined with a small rotation angle of . By placing $\beta\text{=}8^{\circ}$, $g=0.2$, $d_{1}=1.5\times10^{-3}m$,$l=1.2\times10^{-2}m$ into the Eqs. (S40) – (S43), we can obtain the linear transmission coefficients of $t_{xx}$, $t_{xy}$, $t_{yx}$,$t_{yy}$. The complex transmission of circularly polarized waves can be converted from the linear transmission coefficients[6],

$t_{circ}=\left(\begin{matrix} t_{++} & t_{+-} \\ t_{-+} & t_{--} \end{matrix}\right)=\frac{1}{2}\left(\begin{matrix} t_{xx}+t_{yy}+i(t_{xy}-t_{yx}) & t_{xx}-t_{yy}-i(t_{xy}+t_{yx}) \\ t_{xx}-t_{yy}+i(t_{xy}+t_{yx}) & t_{xx}+t_{yy}-i(t_{xy}-t_{yx}) \end{matrix}\right)$ (S44)

The polarization rotation angle is defined as [7]

, (S45)

where $\arg(t_{++})$and $\arg(t_{--})$are the phases of the right-handed circularly polarized wave (RCP), and the left-handed circularly polarized wave (LCP), respectively. The ellipticity $\eta$ is defined as [7]

$\eta=\arctan\left[\left(\left|t_{++}\right|-\left|t_{--}\right|\right)/\left(\left|t_{++}\right|+\left|t_{--}\right|\right)\right]$, (S46)

where $\left|t_{++}\right|$ and $\left|t_{--}\right|$ are the amplitudes of RCP and LCP waves, respectively.

In Fig. 5, we theoretically calculate the linear transmission coefficients，polarization rotation angle and ellipticity of pure chiral meta-atom and FP cavity configuration structures using equations (S40)-(S43) and (S45)-(S46). This indicates that the optical activity effect can be improved using the multiple reflections.

**Note. 4 Mechanism of linearly polarised waves-chiral resonator interaction.**

We have further studied the fundamental mechanism of linearly polarised waves (LPW)-chiral resonator interaction. As presented in Figure S7 (a), the incident y-polarised wave can propagate through the layer A (the grating parallel to x- axis), however x-polarised wave is reflected, under the normal incident linearly polarised wave (LPW). When the y-polarised wave passes through the layer B (the bilayer twisted Cu cross wires), a part of which converts to the x-polarised wave. Consequently, it is divided into four components: co-polarised transmission wave $t_{yy}$, cross-polarised transmission wave $t_{xy}$, co-polarised reflection wave $r_{yy}$, and cross-polarised reflection wave $r_{xy}$. Particularly, the transmission waves $t_{yy}$and $t_{xy}$ can propagate through the layer B, and the waves reflected by the layer B contain the x-polarised ($r_{xy}$) and y-polarised ($r_{yy}$) components. The $r_{yy}$can transmit through the layer A and not contribute to the LPW-chiral resonator interaction, however the $r_{xy}$ is reflected by the layer A to interact with the layer B (chiral resonator). The $r_{xy}$ can be partly converted to the y-polarised wave by the layer B thus containing four parts of $t_{xx}$, $t_{yx}$, $r_{xx}$, and $r_{yx}$. The layer B can transmit the $t_{xx}$ and $t_{yx}$while reflecting the $r_{xx}$and $r_{yx}$, where the $r_{yx}$ propagates through the layer A and the $r_{xx}$ is reflected by the layer A to interact with the layer B. This x-polarised wave will repeat the above process time after time. Namely, the x-polarised wave excited by the incident y-polarised wave can reflect from side to side many times between the layer A and B thus enhancing the OA of the layer B.

Nevertheless, the transmitted waves of the layer B do not contribute to the enhancement of its chirality. To further improve the chiroptical response, we place the grating that is parallel to y-axis (layer C) after the layer B. As presented in Figure S10(b) of SI, the transmitted x-polarized waves in the output of the layer B can propagate through the layer C, while the transmitted y-polarized waves can be reflected to interfere with the layer B and part of which is converted to the x-polarized waves ($t_{xy}$ and $r_{xy}$), however, the rest of which maintain the y-polarized waves ($t_{yy}$and $r_{yy}$). The y-polarized wave can transmit through the layer A, while the x-polarized wave will be reflected to interact with layer B and repeat the process discussed in FigureS10 (a). The $r_{xy}$ can propagate through the layer C and the $r_{yy}$ goes over the process mentioned in Figure S10 (b). Compared to the dual-layered structure schemed in Figure S10(a) where the enhancement of the OA is only caused by the x-polarised wave that repeatedly reflects between layer A and B, in Figure S7 (b) we exploit that by placing layer C, the y-polarized wave can reflect between the layer C and B that in turn further improve the OA. However, it is found that both the y-polarised wave transmitting through the layer A ($t_{yy}$in the layer A of Figure S7(b)) and the x-polarised waves propagating through the layer C (the output of Figure S7(b)) can not contribute to the chirality enhancement. To solve the problem, we replace the pair of orthogonal single layered Cu gratings by the two orthogonal Cu/F4B/Cu trilayered gratings. As shown in Figure S7(c), the two Cu grid films (layer D and E) work as mirrors to reflect the majority of both y- and x- polarised waves that propagate out of the layer A and C, respectively, while still letting a portion of the LPW propagate into the cavity. The reflected y- and x- polarised waves will interact with the layer B and repeat the multi-reflection process discussed in Figure S7(b). This results in a significant improvement of the chiroptical response shown in the right column of Figure1.

**Figure S8.** The enhancement mechanism of the FP resonance in **(a)** dual-layered AB structure **(b)** tri-layered ABC structure **(c)** multi-layered ABCDE structure (Green and red arrows represent y- and x-polarised wave, respectively.)

**Note. 5. The CST simulation of chiral meta-atom in the FP cavity formed by the paired parallel gratings.**

Figure S9(a) schematically shows the chiral molecule placed inside a FP cavity formed by a pair of parallel gratings. As opposed to the FP cavity formed by a pair of orthogonal gratings (Figure 3), there is a significant difference between the two co-polarisation transmission coefficients of $t_{xx}$(black line) and $t_{yy}$(green line), whereas the cross-polarisation transmission coefficient $t_{yx}$(blue line) coincides with $t_{xy}$(red line) (Figure S9(b)).Thus, the rotation angle (optical activity) of the chiral molecule can not be significantly improved inside the cavity (Figure S9(c)).

**Figure S9**. (a) Design schematic, (b) CST simulated transmission coefficients ($t_{xx}$,$t_{yy}$,$t_{yx}$, $t_{xy}$), and (c) rotation angle of a chiral molecule (*θ_1_* = 45° and *θ_2_* = 60°) placed inside a FP cavity formed by two parallel gratings.

**Note. 6. The CST simulation of reflection spectra of handedness-preserving metasurfaces (HPM) and Cu film.**

Figure S10 (a) shows reflection spectra of the co- and cross-polarised components of the HPM mirror. The reflection coefficients of $r_{+-}$ and $r_{- -}$ represent the right-handed circularly polarised (RCP, +) and left-handed circularly polarized (LCP,-) ratio of the reflected power from the HPM to that of the incident LCP wave, respectively. $r_{-+}$ and $r_{++}$represent the LCP and RCP ratio of the reflected power from the HPM mirror for an incident RCP wave, respectively. The top panel presents the conceptual scheme of the HPM mirror. As is seen in the bottom panel, the handedness-preserving reflection ($r_{++}$and $r_{--}$) are much higher than the handedness-reversing reflection ($r_{-+}$ and $r_{+-}$) at 12.6 GHz, where $r_{++}$ overlaps the $r_{--}$, $r_{-+}$ is identical to $r_{+-}$. For a normal metallic mirror, this reflection changes RCP to LCP wave and vice versa, which damages any positive influence of the cavity on the optical activity, as shown in Figure S10 (b).

**Figure S10**. (a) A HPM mirror at a vertical incidence reflects the RCP and LCP waves and preserve their handedness (top panel). Reflection spectra of the co- and cross-polarised components of the HPM (bottom panel). (b) A normal metallic mirror under a vertical incidence reverse the handedness of both RCP and LCP waves in reflection (top panel). Reflection spectra of the co- and cross-polarised components of the normal metallic mirror (bottom panel).

**Note. 7. The CST simulation of chiral meta-atom in the FP cavity formed by a pair of Cu films.**

**Figure S11.** (a) Design schematics and (b) CST simulated rotation angle of the chiral element (*θ_1_* = 45° and *θ_2_* = 60°) placed inside a conventional FP cavity formed by a pair of Cu films.

**Note. 8.** **Chirality enhancement as a function of location inside the FP cavity.**

**Figure S12.** (a) Design schematics of the chiral molecule placed at the various positions inside a FP cavity formed by two metamirrors. (b) The CST simulated roatation angle () spectra at five different positions of *l_1_*=3.25, 4.25, 5.25, 6.25, and 7.25 mm inside the cavity.

**Reference:**

[1] M. Born, E. Wolf, *Principles of Optics: Electromagnetic Theory of Propagation, Interference and Diffraction of Light*, CUP Archive, Elsevier, 2000.

[2] E. Hecht, *Optics, 4th ed*, Addison-Wesley, Reading, MA, 2002.

[3] H. A. Lorentz, *Theory of Electrons*, Dover, 1952.

[4] C. Menzel, C. Rockstuhl, F. Lederer, “Advanced Jones calculus for the classification of periodic metamaterials,” Physical Review A: Atomic, Molecular and Optical Physics, vol. 82, Article ID 053811, 2010.

[5] J. Zhou, D. R. Chowdhury, R. Zhao, et al., “Terahertz chiral metamaterials with giant and dynamically tunable optical activity,” Physical Review B, vol. 86, Article ID 035448, 2012.

[6] J. Zhou, J. Dong, B. Wang, T. Koschny, M. Kafesaki, C. M. Soukoulis, “Negative refractive index due to chirality,” Physical Review B, vol. 79, Article ID 121104, 2009.

[7] Z. Li, H. Caglayan, E. Colak, et al., “Coupling effect between two adjacent chiral structure layers,” Optics Express, vol. 18, no. 6, pp. 5375-5383, 2010.
